# Полиморфизм синдрома Каллера-Джонса

**DOI:** 10.14341/probl13565

**Published:** 2025-12-02

**Authors:** Е. Н. Райкина, А. А. Колодкина, А. В. Болмасова, С. П. Бондаренко, М. С. Панкратова, А. Н. Тюльпаков, К. Г. Забудская, О. Б. Безлепкина

**Affiliations:** Национальный медицинский исследовательский центр эндокринологии им. акад. И.И. ДедоваРоссия; Endocrinology Research CentreRussian Federation; Медико-генетический научный центр имени академика Н.П. Бочкова; Российская детская клиническая больница ФГАОУ ВО РНИМУ им. Н.И. Пирогова Минздрава РоссииРоссия; Research Centre for Medical Genetics; Russian Children’s Clinical HospitalRussian Federation

**Keywords:** синдром Каллера-Джонса, гипопитуитаризм, лицевой дисморфизм, Culler-Jones syndrome, hypopituitarism, facial dysmorphisms

## Abstract

**ОБОСНОВАНИЕ:**

ОБОСНОВАНИЕ. Синдром Каллера-Джонса — редкое аутосомно-доминантное заболевание, причиной которого являются изменения нуклеотидной последовательности в гене GLI2. Распространенность данной патологии неизвестна, так как число наблюдений невелико, у части носителей вариантов в гене GLI2 проявления заболевания отсутствуют. Клинический фенотип заболевания гетерогенен и включает в себя гипопитуитаризм, пороки развития внутренних органов, лицевые дисморфизмы, полидактилию. С момента открытия гена GLI2 Roеssler Е. и соавт. в 2003 г. спектр клинических проявлений, а также понимание патогенеза развития компонентов заболевания значительно расширились. Для гена GLI2 описана неполная пенетрантность, клинический фенотип заболевания различается даже у членов одной семьи с одним и тем же нуклеотидным вариантом.

**ЦЕЛЬ:**

ЦЕЛЬ. Изучение клинического и молекулярно-генетического полиморфизма у пациентов с синдромом Каллера-Джонса.

**МАТЕРИАЛЫ И МЕТОДЫ:**

МАТЕРИАЛЫ И МЕТОДЫ. Проведено одноцентровое неинтервенционное одномоментное несравнительное исследование. Обследованы дети с синдромом Каллера-Джонса с подтвержденной генетической причиной заболевания. Всем пациентам проведено комплексное обследование, включая лабораторно-инструментальные методы диагностики и секвенирование панели генов «Гипопитуитаризм» методом NGS (next-generation sequencing).

**РЕЗУЛЬТАТЫ:**

РЕЗУЛЬТАТЫ. В исследование включены 18 детей (7 девочек; 11 мальчиков) с вариантными заменами в гене GLI2. Возраст на момент обследования составил 8,95 года [4,6; 12,4]. Соматотропная недостаточность установлена у всех детей, возраст диагностики — 2 года [1; 6,5]. Вторичный гипотиреоз диагностирован 13 детям в возрасте 1,5 года [1; 3.5]. Уровень свободного тироксина на момент диагностики — 8,9 пмоль/л [7,5; 11,3]. Вторичный гипокортицизм установлен 10 детям в возрасте 2 лет [1,5; 2,8], уровень кортизола на момент диагностики — 84 нмоль/л [47; 152]. Характерные для синдрома внегипофизарные проявления выявлены у половины пациентов и включают в себя аномалии челюстно-лицевой области, пороки развития сердечно-сосудистой, мочевыделительной систем, а также пороки развития глаз. Полидактилия выявлена у двух детей.

**ЗАКЛЮЧЕНИЕ:**

ЗАКЛЮЧЕНИЕ. Проведенное исследование демонстрирует клинический полиморфизм синдрома Каллера-Джонса, а также отсутствие корреляции «генотип-фенотип» для данного заболевания.

## Abstract (en)

**BACKGROUND:**

BACKGROUND: Culler-Jones syndrome is a rare autosomal dominant disease caused by nucleotide sequence changes in the GLI2 gene. The prevalence of this pathology is unknown, as the number of observations is small, some carriers of variants in the GLI2 gene have no manifestations of the disease. The clinical phenotype of the disease is heterogeneous and includes hypopituitarism, malformations of internal organs, facial dysmorphisms, and polydactyly. Since the discovery of the GLI2 gene by Roessler E. et al. in 2003, the spectrum of clinical manifestations, as well as the understanding of the pathogenesis of the disease components, has expanded considerably. Incomplete penetrance has been described for the GLI2 gene, and the clinical phenotype of the disease differs even among members of the same family with the same nucleotide variant.

**AIM:**

AIM: Study clinical and molecular genetic polymorphism in patients with Culler-Jones syndrome.

**MATERIALS AND METHODS:**

MATERIALS AND METHODS: A single-center, non-interventional, single-stage, non-comparative study was conducted. Children with Culler-Jones syndrome with a confirmed genetic cause of the disease were examined. All patients underwent a comprehensive examination, including laboratory and instrumental diagnostic methods and sequencing (by NGS (next-generation sequencing).

**RESULTS:**

RESULTS: 18 children (7 girls; 11 boys) with variants in the GLI2 gene were included in the study. The age at the time of examination was 8.95 years [4,6; 12.4]. Growth hormone deficiency was noted in all children at age of 2 years [1; 6,5]. Central hypothyroidism was diagnosed in 13 children at the age of 1.5 years [1; 3.5]. Free thyroxine level at the time of diagnosis was 8.9 pmol/L [7.5; 11.3]. Secondary hypoadrenocorticism was diagnosed in 10 children at the age of 2 years [1.5; 2.8], with a cortisol level of 84 nmol/L at the time of diagnosis [47; 152]. Extrahypophyseal manifestations characteristic of the syndrome were detected in half of the patients and included maxillofacial anomalies, malformations of the cardiovascular and urinary systems and eye malformations. Polydactyly was detected in two children.

**CONCLUSION:**

CONCLUSION: The present study demonstrates the clinical polymorphism of Culler-Jones syndrome and the lack of genotype-phenotype correlation for this disease.

## ОБОСНОВАНИЕ

Синдром Каллера-Джонса — редкое аутосомно-доминантное заболевание, развивающееся вследствие вариантных замен в гене GLI2. Синдром характеризуется выраженной вариабельностью клинического фенотипа. Характерен гипопитуитаризм — от изолированного дефицита гормона роста до множественного дефицита гормонов гипофиза. Описана ассоциация с полидактилией, лицевыми дисморфизмами, умственной отсталостью, аномалиями гипоталамо-гипофизарной области, пороками развития внутренних органов [1][2]. Все описания пациентов с синдромом Каллера-Джонса в мировой литературе свидетельствуют о неполной пенетрантности и высокой вариабельности экспрессии гена GLI2, что, в частности, связывают с влиянием сочетания множества факторов — экологических, генетических (например, варианты в других локусах или дигенное наследование) [2].

GLI2 (OMIM 165230) — это ген семейства GLI-Kruppel, картированный на длинном плече 2 хромосомы (2q14.2) [2]. Свое название GLI2 получил от глиомы головного мозга, в ткани которой впервые была выявлена повышенная экспрессия данного гена [3]. Кодируемый геном одноименный белок представляет собой фактор транскрипции цинковых пальцев, обладающий транскрипционно-репрессивной активностью на амино-конце и транскрипционно-активирующей активностью на карбоксильном конце [4] .

GLI2 является членом семейства белков GLI (включает также GLI1 и GLI3), которые участвуют в сигнальном пути Sonic Hedgehog (SHH), регулирующем дифференцировку, пролиферацию и полярность стволовых клеток хорды и дна пластинки в развивающемся спинном мозге [3]. Помимо этого, GLI2 играет решающую роль в развитии промежуточного мозга и дистальных отделов конечностей во время эмбриогенеза [5].

Ген GLI2 участвует в развитии гипофиза, что впервые было показано на мышиной модели: у эмбрионов мышей вследствие аберраций в гене GLI2 отмечалась гипоплазия аденогипофиза [6].

Culler F. и Jones K. первыми в 1984 г. описали когорту людей с триадой признаков: полидактилия, гипопитуитаризм и дисморфические черты лица (гипоплазия средней части лица, гипоплазия спинки носа, широко расставленные ноздри, широкие надбровные дуги, гипотелоризм глаз) с аутосомно-доминантным типом наследования [7]. Результаты многолетних исследований позволили доказать, что синдром Каллера-Джонса ассоциирован с гетерозиготными вариантами в гене GLI2, а также значительно расширить представление о фенотипе синдрома и включить в него ряд других признаков (рис. 1). Все симптомы при данном синдроме можно разделить на две большие группы [4][8–11]:

- ассоциированные с гипопитуитаризмом: дефицит тропных гормонов гипофиза, изменения гипофиза по данным магнитно-резонансной томографии головного мозга (синдром «прерывания» ножки гипофиза), гипогликемии (и ассоциированная с ними задержка психомоторного развития), холестаз, микропения, крипторхизм;

- пороки других органов: дефекты срединной линии (расщелины верхней губы, твердого и мягкого неба), дисморфические черты лица, микроцефалия, голопрозэнцефалия (ГПЭ), эпилепсия, аномалии развития сердечно-сосудистой и мочевыделительной систем, пороки развития зрительного аппарата.

Гетерозиготные варианты в гене GLI2 могут наследоваться по аутосомно-доминантному типу или возникать de novo (51% по материнской линии, 40% по отцовской и 9% de novo) [5].

**Figure:**
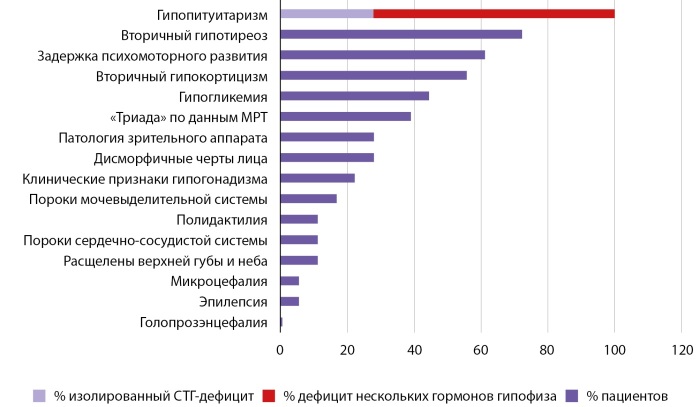
Рисунок 1. Частоты встречаемости симптомов, характерных для синдрома Каллера-Джонса, среди исследуемых пациентов.

## ЦЕЛЬ ИССЛЕДОВАНИЯ

Изучение клинического полиморфизма у пациентов с синдромом Каллера-Джонса.

## МАТЕРИАЛЫ И МЕТОДЫ

## Место и время проведения исследования

Исследование проведено у пациентов, проходивших стационарное и/или амбулаторное обследование в Институте детской эндокринологии ФГБУ «НМИЦ эндокринологии» Минздрава России за период с сентября 2015 по декабрь 2024 гг.

## Изучаемые популяции (одна или несколько)

Популяция: 18 пациентов с вариантными аберрациями в гене GLI2.

Критерий включения: пациенты с подтвержденными генетически синдромом Каллера-Джонса (изменения нуклеотидной последовательности в гене GLI2).

Критерий исключения: пациенты с вариантными аберрациями в других генах, ассоциированных с врожденными формами гипопитуитаризма.

Способ формирования выборки из изучаемой популяции (или нескольких выборок из нескольких изучаемых популяций): сплошной.

## Дизайн исследования

Одноцентровое одномоментное несравнительное исследование.

## Методы

Протокол исследования включал в себя сбор клинико-анамнестических данных, физикальное обследование с оценкой физического и полового развития, оценку жалоб, подробный сбор анамнеза жизни и заболевания, данных наследственного анамнеза.

Лабораторные исследования проводились всем пациентам в клинико-диагностической лаборатории ФГБУ «НМИЦ эндокринологии» Минздрава России на автоматическом иммунохемилюминесцентном анализаторе Vitros 3600 Ortho Clinical Diagnostics, «Johnson& Johnson», США, и включали в себя оценку уровня инсулиноподобного фактора роста 1 (ИФР-1), тиреотропного гормона (ТТГ), свободного тироксина (свТ4), пролактина (ПРЛ), кортизола. У девочек старше 13 лет и мальчиков старше 14 лет исследовались уровни лютеинизирующего (ЛГ) и фолликулостимулирующего (ФСГ) гормонов, тестостерона, эстрадиола.

Магнитно-резонансная томография головного мозга проводилась на аппарате Optima MR450w (GE Healthcare) c напряженностью магнитного поля 1,5 Тесла. Исследование проводилось в Т1- и Т2-взвешенных режимах по стандартной методике. Описание сделанных на базе других лечебно-профилактических учреждений исследований проводилось при предоставлении цифровых носителей.

Молекулярно-генетическое исследование проводилось в ФГБУ «НМИЦ эндокринологии» Минздрава России. Проведено секвенирование ДНК лимфоцитов периферической крови на приборе MiSeq (Illumina, USA) методом парно-концевых чтений с применением авторской панели «Гипопитуитаризм», содержащей праймеры для мультиплексной ПЦР и секвенирования кодирующих последовательностей следующих 23 генов: ACAN, ARNT2, DLK1, DRD2, FOXA2, GH1, GHRH, GHRHR, GHSR, GLI2, HESX1, IGSF1, LHX3, LHX4, OTX2, PAX6, POU1F1, PROP1, RNPC3, SHH, SOX2, SOX3, TBX19. Оценка патогенности вариантов нуклеотидной последовательности проводилась согласно международным и российским рекомендациям [11][12].

## Статистический анализ

Расчет данных производился с помощью статистического пакета Statistica 8 (StatSoft inc., США), MS Exсel 2016 (Microsoft, США). Количественные результаты представлены в виде медианы (Ме) и квартилей [ Q1; Q3], соответствующих 25 и 75 перцентилям. 95-процентный доверительный интервал (ДИ) для относительных частот рассчитан с помощью метода Клоппера–Пирсона с использованием интернет-калькулятора https://www.graphpad.com/quickcalcs/confInterval1.

## Этическая экспертиза

Проведение исследования одобрено локальным этическим комитетом ФГБУ «НМИЦ эндокринологии» Минздрава России. Протокол №16 от 13.09.2023 года.

## РЕЗУЛЬТАТЫ

В исследование включены 18 детей с вариантными заменами в гене GLI2 (7 девочек; 11 мальчиков), возраст на момент обследования — 8,5 года [ 4,6; 12,4]. Выявленные варианты в гене GLI2 ранее не были описаны в мировой литературе. У 7 пациентов (38,9%) варианты оценивались как вероятно патогенные, у 11 пациентов (61,1%) — как варианты с неопределенной клинической значимостью.

Магнитно-резонансная томография проведена 13 детям. У 11 пациентов (84,6%) были выявлены различные пороки гипоталамо-гипофизарной области:

1) синдром «прерывания» ножки гипофиза (классическая триада) — у 7 детей (53,8%);

2) гипоплазия аденогипофиза — у 2 детей (15,4%);

3) септооптическая дисплазия — у 1 ребенка (7,7%);

4) эктопия нейрогипофиза — у 1 ребенка (7,7%);

5) отсутствие патологии гипоталамо-гипофизарной области — у 2 детей (15,4%).

В таблице 1 представлена подробная характеристика пациентов с синдромом Каллера- Джонса.

**Table:** Таблица 1. Клинические и генетические данные пациентов с синдромом Каллера-Джонса * Нумерация кодирующей последовательности гена GLI2 дана по референсу NM_001374353.1 (MANE Select транскрипт).

№	Возраст пациента	МРТ головного мозга/гипофиза	Дефицит гормонов гипофиза	Аномалии конечностей	Задержка психомоторного развития	Пороки челюстно-лицевой области	Пороки мочевыделительной системы	Пороки сердечно-сосудистой системы	Аномалии развития зрительного аппарата	Другие клинические и фенотипические особенности	Вариант в гене GLI2*
1	15 лет	Нет данных	СТГ	Нет	Да Гипогликемий не было	Нет	Нет	Нет	Миопический астигматизм	Микроцефалия	c.4049G>T p.(Gly1350Val) вариант с неопределенной клинической значимости, ранее не описан
2	11 лет	Гипоплазия аденогипофиза, аплазия ножки гипофиза, эктопия нейрогипофиза	СТГ, ТТГ, АКТГ	Нет	Да Гипогликемии в неонатальном периоде	Нет	Нет	Нет	Сходящееся содружественное косоглазие, миопический астигматизм	Гипогликемические судороги	c.1860_1861del p.(Glu621GlyfsTer40) вероятно патогенный, ранее не описан
3	10,5 лет	Перивентрикулярная лейкомаляция в затылочных долях обеих сторон	СТГ	Нет	Да Гипогликемии в анамнезе, перинатальное поражение ЦНС	Нет	Умеренная пиелоэктазия левой почки	Нет	Нет	Гипогликемии в анамнезе, правосторонняя кривошея	c.1431_1432del p.(Arg478AlafsTer41) вероятно патогенный, ранее не описан
4	15 лет	Нет данных	СТГ	Нет	Нет данных	Нет	Нет	Нет	Нет	Нет данных	c.1150C>G p.(Leu384Val) вариант с неопределенной клинической значимостью ранее не описан
5	3,5 года	Гипоплазия аденогипофиза и воронки гипофиза, эктопия нейро-гипофиза	АКТГ, СТГ, ТТГ, пролактин	«Скученность» пальцев кистей и стоп	Да Гипогликемии в неонатальном периоде	Дисморфичные черты лица, гипоплазированная спинка носа, широко расставленные ноздри, гипотелоризм	Нет	Состояние после перевязки открытого артериального протока, открытое овальное окно, умеренная гипоплазия фиброзного кольца трикуспидального клапана и левой легочной артерии	Нет	Гипогликемии, эпилепсия	c.2690G>A p.(Arg897Gln) вариант с неопределенной клинической значимостью ранее не описан
6	6,5 лет	Гипоплазия аденогипофиза и воронки, эктопии нейрогипофиза	СТГ, ТТГ	Нет	Да Хроническая внутриутробная гипоксия плода	Умеренно дисморфичные черты лица, широко расставленные ноздри, уплощение средней части лица	Нет	Нет	Нет	Гипоспадия	с.2907_2923del p.(Phe970ArgfsTer38) вероятно патогенный, ранее не описан
7	3 года	Гипоплазия аденогипофиза и воронки, эктопии нейрогипофиза	СТГ, АКТГ, ТТГ	Нет	Да Гипогликемии в неонатальном периоде	Нет	Нет		Миопический астигматизм	Крипторхизм	c.2306del p.(Pro769ArgfsTer11) вероятно патогенный, ранее не описан
8	2 года	Не проводилось	СТГ, АКТГ, ЛГ, ФСГ, ТТГ	Нет	Да Гипогликемии в неонатальном периоде	Двухсторонняя расщелина верхней губы и нёба, гипоплазия спинки носа, широко расставленные ноздри	Нет	Нет	Нет	Гипогликемии, микропения, двусторонний крипторхизм	c.1478A>G, p.His493Arg вариант с неопределенной клинической значимостью, ранее не описан
9	8 лет	Гипоплазия аденогипофиза и воронки, эктопии нейрогипофиза	СТГ, АКТГ, ТТГ, пролактин	Нет	Нет	Нет	Нет	Нет	Миопический астигматизм	Гипогликемии	c.1495C>T p.(Arg499Cys) вариант с неопределенной клинической значимостью, ранее не описан
10	9 лет	Норма	СТГ	Нет	Нет	Нет	Нет	Нет	Нет	Ограничение поворота шеи вправо, укорочение шеи	c.3998G>T p.(Gly1333Val) вариант с неопределенной клинической значимостью, ранее не описан
11	14 лет	Гипоплазия аденогипофиза	СТГ, ТТГ, АКТГ, ЛГ/ФСГ	Нет	Да Гипогликемия и дыхательные нарушения в неонатальном периоде	Нет	Нет	Нет	Нет	Нет	c.1384C>T p.(Arg462Trp) вариант с неопределенной клинической значимостью, ранее не описан
12	3 года	Септооптическая дисплазия	СТГ, ТТГ, АКТГ	Аномалия развития верхней конечности	Да, Гипогликемии в неонатальном периоде	Нет	Нет	МАРС: ООО	Гипоплазия зрительного нерва	ДЦП атонически-астатическая форма	c.3998G>T p.(Gly1333Val) вариант с неопределенной клинической значимостью, ранее не описан
13	8 лет	Эктопия нейрогипофиза	СТГ, ТТГ	Нет	Нет, гипогликемий не было	Нет	Нет	Нет	Нет	Эпикант, гипотелоризм, низкорасположенные ушные раковины, треугольный подбородок, скошенный затылок	c.1622A>T p.(His541Leu) вариант с неопределенной клинической значимостью, ранее не описан
14	13 лет	Гипоплазия аденогипофиза, признаки участка кистозной транформации в левой гемисфере мозжечка, последствия перенесенного ОНМК — по геморрагическому типу	СТГ	Нет	Нет, гипогликемий не было	Нет	Нет	Нет	Нет	Нейрофиброматоз 1 типа. Сирингомиелия Th3-Th10. Начальная дорсальная деформация дисков L4-L5, L5-S1	c.3998G> T p.(Gly1333Val) вариант с неопределенной клинической значимостью, ранее не описан
15	11 лет	Гипоплазия аденогипофиза и воронки, эктопии нейрогипофиза	СТГ, ТТГ, АКТГ	Нет	Нет	Нет	Нет	Нет	Нет	Микропения, венечная гипоспадия	c.1699C>T р.Нis5677Tyr вероятно патогенный, ранее не описан
16	13 лет	Гипоплазия аденогипофиза и воронки, эктопии нейрогипофиза	СТГ, АКТГ, ТТГ, ЛГ/ФСГ	Нет	Нет	Нет	Нет	Нет	Нет	Гипогликемии в неонатальном периоде	c.3628C>T p.(Gln1210Ter) вероятно патогенный, ранее не описан
17	11 мес.	Не проводилось. По результатам нейросонографии патологии не выявлено	СТГ, ТТГ	Полидактилия обеих кистей, стопы слева	Да. Грубая задержка темпов моторного развития. Легкая задержка психо-предречевого развития. Гипогликемии в неонатальном периоде	Умеренно дисморфичные черты лица, широко расставленные ноздри	Пиелоэктазия справа	Нет	Нет	Гипогликемии в неонатальном периоде, микропения	1. в гене GLI2 c.2994_3021del p.(Asp999ArgfsTer105) вероятно патогенный, ранее не описан 2. в гене LHX4 c.180271896G>A, c.668G>A p.(Arg223His) вероятно патогенный, ранее не описан
18	5 лет	Не проводилось	СТГ, АКТГ, ТТГ, ЛГ/ФСГ	Преаксиальная полидактилия правой кисти	Нет	Умеренно дисморфичные черты лица, нарушение зубного ряда, гипотелоризм глаз, расщелина твердого и мягкого неба (оперирован в 2020 г.)	Агенезия левой почки, гидронефроз правой почки	Нет	Нет	Гипогликемии, судороги на фоне ОРВИ, микропения	c.148+44465C>T вариант с неопределенной клинической значимостью, ранее не описан

Клинические проявления гипопитуитаризма у детей из нашей когорты варьировали от изолированного дефицита гормона роста до множественного дефицита гормонов гипофиза.

Соматотропная недостаточность установлена у всех детей (95% ДИ [83; 100]). Возраст диагностики — 2 года [ 1; 6,5], уровень ИФР-1 на момент диагностики — 15 нг/мл [ 8,9; 25,6]. Динамика ростовых показателей пациентов на фоне терапии соматропином представлена в таблице 2.

**Table:** Таблица 2. Динамика роста на терапии соматропином у пациентов с синдромом Каллера-Джонса

№	Возраст пациента	Возраст начала терапии соматропином	SDS роста до терапии	Доза соматропина мг/кг/сутки	Длительность терапии	Суммарная прибавка в росте за период терапии, см	ΔSDS роста
1	15 лет	12,6 года	-2,4	0,033	1 год 5 мес	10	0,9
2	11 лет	1,5 года	-3,0	0,026	10 лет	48	4,28
3	10,5 года	3,6 года	-3,84	0,033	6 лет	47,6	2,88
4	15 лет	2,1 года	-1,87	0,033	2,5 года	8	2,54
5	3,5 года	0,6 года	-1,82	0,025	3,5 года	20,5	2,36
6	7,5 года	6 лет	-2,5	0,033	2 года	-	-
7	3 года	1,5 года	-3,54	0,02	2,5 года	19	3,23
8	2 года	0,2 года	-2,08	0,035	2,5 года	-	-
9	8 лет	1,9 года	-5,2	0,033	7 лет	55,9	2,13
10	9 лет	8 лет	-3,11	0,033	1 год	-	-
11	14 лет	2,9 года	-2,53	0,033 ->0,008	11 лет	80,2	3,49
12	3 года	3 года	-1,96	0,033	2 мес	-	-
13	7 лет	6,6 года	-3,38	0,033	1 год 4 мес	14,5	1,51
14	13 лет	9,4 года	-3,11	0,033	3 года 9 мес	23,5	1,09
15	11 лет	3,4 года	-3,3	0,033	7 лет 6 мес	57,7	3,31
16	13 лет	1,9 года	-2,6	0,033	11 лет	-	3,37
17	11 мес	0,9 года	-3,06	0,033	5 мес	-	-
18	5 лет	4,9 года	-5,58	0,033	2 мес	-	-

Тиреотропная недостаточность диагностирована 13 детям (95% ДИ [ 46; 90]) в возрасте 1,5 лет [ 1; 3,5]. Уровень свободного тироксина на момент диагностики — 8,9 пмоль/л [ 7,5; 11,3].

Вторичный гипокортицизм установлен 10 детям (95% ДИ [ 31; 78]) в возрасте 2 лет [ 1,5; 2,8], уровень кортизола на момент диагностики — 84 нмоль/л [ 47; 152].

Дефицит антидиуретического гормона не был выявлен ни у одного пациента.

У 11 пациентов (95% ДИ [ 35; 82]) диагностирована задержка психомоторного развития. У 8 из них (95% ДИ [ 21; 69]) отмечались гипогликемии в анамнезе, что могло стать причиной отставания в психомоторном развитии. У 3 пациентов эпизоды гипогликемии в неонатальном периоде не фиксировались.

Аномалии челюстно-лицевой области отмечены у 5 детей (95% ДИ [ 9,7; 53]). У двух из них дисморфичность развития характеризовалась расщелинами верхней губы и неба.

У пациентов 3, 17 и 18 клиническая картина характеризуется врожденным пороком мочевыделительной системы (95% ДИ [ 13,5; 41]) в виде пиелоэктазии почки, у 18 пациента также отмечается агенезия левой почки.

Пороки сердечно-сосудистой системы выявлены у 2 пациентов (95% ДИ [ 1,4; 34]). Сопутствующая кардиологическая патология потребовала хирургического вмешательства и включала открытый артериальный проток, открытое овальное окно, умеренную гипоплазию фиброзного кольца трикуспидального клапана и левой легочной артерии у пациентки 5. Гемодинамически незначимый порок (открытое овальное окно) выявлен у пациента 12.

Офтальмологическая патология выявлена у 5 детей (95% ДИ [ 9,7; 53]). Миопический астигматизм установлен 4 детям, у одного из них сочетается со сходящимся содружественным косоглазием. У одной пациентки имеет место гипоплазия зрительных нервов.

Клинические признаки гипогонадотропного гипогонадизма на первом году жизни отмечены у 4 детей (95% ДИ [ 6,4; 47,6]) и включали в себя крипторхизм и микропению. У одного ребенка клиническая картина характеризовалась также венечной гипоспадией.

Характерный для синдрома Каллера-Джонса фенотипический признак (полидактилия) был выявлен у пациента 17 с вероятно патогенным вариантом и пациента 18 с вариантом неопределенного клинического значения (95% ДИ [ 1,4; 34]) в гене GLI2.

## ОБСУЖДЕНИЕ

Полученные в ходе исследования результаты демонстрируют клинический полиморфизм синдрома Каллера-Джонса, что совпадает с данными мировой литературы [5][9][14]. Анализ клинических данных показал отсутствие корреляции фенотипических проявлений с патогенностью вариантов гена GLI2 у наших пациентов. У пациента 2 с вероятно патогенным вариантом в гене диагностированы дефициты СТГ, АКТГ и ТТГ, но отсутствовали полидактилия, дисморфические черты лица и другие пороки внутренних органов. Большинство пациентов с вариантами неопределенного клинического значения имеют множественный дефицит гормонов гипофиза в сочетании с пороками развития других органов (табл. 1).

Важно отметить, что GLI2 является геном с частыми нормальными вариациями. Z-score в базе данных gnomAD v4.0.0 составляет 0,49, что указывает на высокую толерантность к миссенс-вариантам.

Патогенные варианты в гене GLI2 могут быть обнаружены у здоровых людей в связи с неполной пенетрантностью гена, однако ее процент в настоящее время неизвестен. Неполная пенетрантность также свидетельствует о том, что для формирования фенотипа, вероятно, необходимо наличие других генетических факторов и факторов окружающей среды [2]. Среди всех пациентов только у пациента 17 была выявлена дополнительная, помимо гена GLI2, аберрация в гене LHX4, гетерозиготные варианты в котором также приводят к развитию гипопитуитаризма. Однако полидактилия, выявленная у пациента, характерна для синдрома Каллера-Джонса и не встречается среди пациентов с аберрациями гена LHX4.

В различных исследованиях случаи пациентов с идентичными вариантами в гене GLI2 приводились в качестве доказательства его неполной пенетрантности и вариабельной экспрессии [9][15][16].

В литературном обзоре Arnhold I. и соавт. проанализированы публикации клинических случаев пациентов с гипопитуитаризмом и наличием аберраций в гене GLI2. В исследование была включена группа, состоящая из 25 пациентов из 16 семей с вероятно патогенными вариантами. Среди 11 родителей, которые являются носителями такого же варианта, как у их детей, у двух выявлен гипопитуитаризм (один из них с полидактилией), у трех клиническая картина характеризовалась изолированной полидактилией, а у шестерых какие-либо симптомы отсутствовали. Интересно, что среди 12 пациентов с одним и тем же вариантом гена GLI2 у 10 человек из одной семьи отмечалась полидактилия, тогда как у 2 человек из другой семьи данный признак отсутствовал. У шести выявлен гипопитуитаризм — изолированный дефицит гормона роста у двух человек, у четырех — множественный дефицит гормонов гипофиза. У 2 родственных пациентов выявлены такие фенотипические особенности, как одиночный срединный резец, у одного из них также отмечается расщелина верхнего неба. У 10 пациентов из другой семьи данных особенностей выявлено не было [2]. Все это подтверждает то, что даже при самых тяжелых вариантах в гене пенетрантность GLI2 является неполной.

Среди родителей пациентов из нашей когорты молекулярно-генетическое исследование было проведено у матери 1 пациента, выявлен аналогичный вариант с неопределенной клинической значимостью. Женщина не обследована на предмет гипопитуитаризма, однако ее рост ниже среднего популяционного и составляет 150 см. Из наследственного анамнеза также известно, что родной дядя и дедушка по материнской линии были прооперированы по поводу полидактилии, рост обоих при этом 185 см.

У матери 14 пациента выявлен такой же вероятно патогенный вариант в гене GLI2, как и у ребенка. Из клинических проявлений у женщины отмечается низкорослость и полидактилия. У отца 18 пациента был выявлен такой же вариант в гене GLI2, как и у ребенка, однако у него отсутствовали какие-либо фенотипические проявления синдрома Каллера-Джонса.

Ранее варианты в гене GLI2 ассоциировали с фенотипом ГПЭ. ГПЭ проявляется структурными аномалиями срединной линии и дефектами разделения на полушария переднего мозга [17]. В настоящий момент доказано, что ГПЭ при аберрациях в гене GLI2 встречается редко. Самая большая когорта пациентов с вариантами в гене GLI2 проанализирована в исследовании Bear и соавт. Из 112 пациентов только у 1 (0,9%) пациента с патогенным вариантом в гене GLI2 обнаружена ГПЭ [1]. В нашем исследовании ГПЭ не описана ни у одного пациента.

В настоящее время факторы, влияющие на клинический полиморфизм синдрома Каллера-Джонса, недостаточно изучены в силу небольшого количества зарегистрированных клинических случаев, отсутствия достаточной осведомленности врачей о заболевании, ограниченных возможностей для изучения влияния эпигенетических факторов, влияющих на фенотип пациентов.

## Ограничения исследования

В ходе исследования могли возникнуть смещения результатов по причине недостаточного объема выборки, неопределенность оценок также связана с малым размером исследуемой выборки.

## ЗАКЛЮЧЕНИЕ

Синдром Каллера-Джонса является примером сочетания гипопитуитаризма с неэндокринными патологиями и связан с вариантами в гене GLI2, участвующем в раннем эмбриогенезе. Наличие пороков развития внутренних органов в сочетании с гипопитуитаризмом является показанием к проведению молекулярно-генетического исследования у данных пациентов с целью выявления других возможных компонентов синдрома. Необходимы дальнейшие исследования с целью поиска возможных причин вариабельности фенотипических проявлений и разработки персонализированного медико-генетического консультирования семей с вариантами гена GLI2.

## ДОПОЛНИТЕЛЬНАЯ ИНФОРМАЦИЯ

Источники финансирования. Работа проведена в рамках темы госзадания 123021000045–4 «Генетическая персонификация редких вариантов задержки роста и полового развития у детей».

Конфликт интересов. Авторы декларируют отсутствие явных и потенциальных конфликтов интересов, связанных с содержанием настоящей статьи.

Участие авторов. Все авторы одобрили финальную версию статьи перед публикацией, выразили согласие нести ответственность за все аспекты работы, подразумевающую надлежащее изучение и решение вопросов, связанных с точностью или добросовестностью любой части работы.
